# Motor fatigue is associated with asymmetric connectivity properties of the corticospinal tract in multiple sclerosis

**DOI:** 10.1016/j.nicl.2020.102393

**Published:** 2020-08-25

**Authors:** Christian Bauer, Tim B. Dyrby, Finn Sellebjerg, Kathrine Skak Madsen, Olivia Svolgaard, Morten Blinkenberg, Hartwig Roman Siebner, Kasper Winther Andersen

**Affiliations:** a Danish Research Centre for Magnetic Resonance, Centre for Functional and Diagnostic Imaging and Research, Copenhagen University Hospital Hvidovre, Denmark; b Radiography, Department of Technology, University College Copenhagen, Copenhagen, Denmark; c Department of Neurology, Copenhagen University Hospital Bispebjerg, Denmark; d Institute of Clinical Medicine, Faculty of Health and Medical Sciences, University of Copenhagen, Copenhagen, Denmark; e Danish Multiple Sclerosis Center, Rigshospitalet, University of Copenhagen, Copenhagen, Denmark; f Department of Applied Mathematics and Computer Science, Technical University of Denmark, Kongens Lyngby, Denmark

**Keywords:** Anatomical connectivity mapping, Left-right asymmetry, Corticospinal tract, Lateralization index, Diffusion weighted imaging, Motor fatigue, Multiple sclerosis (MS)

## Abstract

Multiple Sclerosis (MS) is characterized by demyelination and neurodegeneration of the central nervous system and causes excessive fatigue in more than 80% of the patients. The pathophysiologic mechanisms causing fatigue are still largely unknown. In 46 right-handed patients with relapsing-remitting MS and 25 right-handed controls, we performed diffusion MRI and applied streamline based probabilistic tractography to derive unilateral anatomical connectivity maps for the white matter of the right and left hemispheres. The maps provide an indication how often a streamline has passed through a given voxel. Since tractography based anatomical connectivity mapping (ACM) is sensitive to disease-induced changes in anatomical connectivity, we used ACM to test whether motor fatigue is associated with altered ipsi-hemispherical anatomical connectivity in the major motor output pathway, the corticospinal tract (CST). Patients had higher mean ACM values in the CST than healthy controls. This indicated that a higher number of streamlines, starting from voxels in the same hemisphere, travelled through the CST and may reflect an accumulated disease-induced disintegration of CST. The motor subscale of the Fatigue Scale for Motor and Cognitive functions (FSMC_MOTOR_) was used to define sub-groups with (n = 29, FSMC_MOTOR_ score ≥ 27) and without motor fatigue (n = 17, FSMS_MOTOR_ score ≤ 26). Patients without fatigue only showed higher ACM values in right CST, while mean ACM values were unaltered in left CST. The higher the mean ACM values in the left relative to the right CST, the more patients reported motor fatigue. Left-right asymmetry in anatomical connectivity outside the CST did not scale with individual motor fatigue. Our results link lateralized changes of tractography-based microstructural properties in the CST with motor fatigue in relapsing-remitting MS.

## Highlights

• Anatomical connectivity mapping measures voxel-wise anatomical connectivity mapping (ACM) of cerebral white-matter.
• ACM of corticospinal tract (CST) was studied in relapsing-remitting multiple sclerosis.
• Patients with motor fatigue show a left-right asymmetry in AC in CST.
• The higher ACM in left relative to right CST, the more severe is motor fatigue.

## 1 Introduction

Multiple sclerosis (MS) is an inflammatory disease of the central nervous system causing widespread axonal demyelination and degeneration, which can be mapped by magnetic resonance imaging (MRI) (Filippi et al., 2019). Fatigue is a very common and highly disabling symptom in MS. More than 80% of the patients are affected by a marked reduction of physical and mental energy (Induruwa et al., 2012, Penner and Paul, 2017). Fatigue can either manifest as effort-independent fatigue (i.e., state fatigue) leading to a general sensation of fatigue or effort dependent fatigue (i.e., fatigability) provoked by physical or mental effort (Kluger et al., 2013). Fatigue is either defined as a primary or secondary phenomenon. Primary fatigue is directly caused by disease processes, whereas secondary factors, such as disease-related pain, reduced sleep quality, depression, medication effects, reduced physical activity, physical impairment and other medical diseases, contribute to the magnitude of experienced fatigue (Kos et al., 2008, Penner et al., 2007). Several disease-related processes are thought to contribute to fatigue in MS, including proinflammatory cytokines (Gold, 2006), endocrine effects (Gottschalk et al., 2017), as well as structural (Bermel and Bakshi, 2006, Riccitelli et al., 2011) and functional alterations in the central nervous system (Benwell et al., 2007). Yet, it still remains a challenge to determine specific mechanisms at the system level that can explain why an individual patient experiences fatigue and others do not (Induruwa et al., 2012).

Conventional structural MRI methods, including T1-weighted, T2-weighted and fluid-attenuated inversion recovery (FLAIR) imaging, have been used to identify structural brain correlates of fatigue. So far, structural MRI studies have focused on brain volume loss, lesion load and lesion location, yielding inconsistent results (Krupp et al., 2010, Pardini et al., 2010, Tedeschi et al., 2007). Cortical grey matter loss in the posterior parietal lobe was proposed as structural marker of fatigue (Amato and Portaccio, 2012), while others reported a relation between local atrophy in the frontal lobes or deep grey matter nuclei with fatigue (Bermel and Bakshi, 2006, Riccitelli et al., 2011).

Diffusion MRI (dMRI) has also been used to probe microstructural correlates of fatigue in MS. Diffusion tensor imaging (DTI) yields tensor-based metrics of the regional diffusion of water, such as fractional anisotropy (FA) and mean diffusivity (MD) (Budde et al., 2008). These metrics are widely used because they are highly sensitive to microstructural changes, but they have limited specificity in terms of the underlying tissue alteration (Budde et al., 2008). Several DTI studies reported an association between microstructural changes in several white-matter fibre bundles and deep grey matter nuclei and fatigue in patients with MS (Bester et al., 2013, Genova et al., 2013, Gobbi et al., 2014, Pardini et al., 2015, Pardini et al., 2010, Rocca et al., 2014, Sander et al., 2016, Wilting et al., 2016). However, other studies were not able to show a link between microstructural DTI measures and fatigue (Andreasen et al., 2010, Bisecco et al., 2016, Codella et al., 2002, Hanken et al., 2016). Taken together, the existing body of structural MRI studies has so far failed to pinpoint regional structural correlates of fatigue in patients with MS. The failure to identify a consistent local structural abnormality may be due to the pathophysiology of fatigue, which may arise from diffuse rather than focal pathology. For instance, fatigue might result from more widespread changes in structural brain connectivity even along the same tract rather than from a focal structural change confined to a specific brain region.

MRI-derived measures reflecting changes in anatomical connectivity might be more appropriate to identify brain correlates of fatigue in MS. Diffusion sensitive magnetic resonance imaging (dMRI) of cerebral white matter can yield a voxel-wise estimate of structural connectivity using a tractography-based method called anatomical connectivity mapping (ACM) (Cercignani et al., 2012). Using each voxel as a seeding source, ACM repeatedly applies probabilistic tractography from each white matter voxel and generates voxel-specific estimates of the number of streamlines that pass a given voxel (Cercignani et al., 2012, Liptrot et al., 2014). A voxel with a high ACM value is a voxel that is part of a high number of streamlines. Conversely, a voxel with a low ACM value is a voxel that belongs only to a few streamlines. Moreover, since tractography maps individual tract systems the voxel-wise streamline count express a system specific anatomical connectivity of the tracts passing through it (Liptrot et al., 2014).

Tractography-informed ACM has been applied to identify disease-related changes in white matter connectedness in blind individuals (Reislev et al., 2016), Alzheimeŕs disease (Bozzali et al., 2011) and in patients with MS (Bozzali et al., 2013, Lyksborg et al., 2014). In MS, ACM identified regional changes in brain connectivity reflecting motor or cognitive dysfunction, which differed in spatial location and extent from alterations of standard DTI metrics such as fractional anisotropy (FA) and mean diffusivity (MD)(Bozzali et al., 2013, Lyksborg et al., 2014).

In this study, we employed ACM in mildly affected patients with relapsing-remitting MS and healthy controls to test the hypothesis that motor fatigue is associated with altered ACM of the corticospinal tract (CST). Here, we applied the constrained spherical deconvolution method for probabilistic tractography to generate ACM. To study the unilateral ACM of the left and right CST, tractography was restricted to either the right half or left half of the brain by excluding callosal streamline projections. Using the motor subscale of the Fatigue Scale for Motor and Cognitive functions (FSMC_MOTOR_) (Penner et al., 2009), we tested for a relationship between the individual expression of motor fatigue and lateralized changes in the ACM of CST and compared with DTI metrics e.g. FA and MD.

## 2 Materials and methods

### 2.1 Participants

Fifty right-handed patients with relapsing-remitting MS aged 18–25 years and 25 age and sex-matched healthy individuals were included in the present cross-sectional study. The study was conducted at Danish Research Centre for Magnetic Resonance (DRCMR), Hvidovre Hospital in the Capital Region of Copenhagen. Patients were recruited at the Danish Multiple Sclerosis Center, Rigshospitalet, University of Copenhagen, or via the Danish Multiple Sclerosis Society. Patients were eligible to participate in the study if they were diagnosed with relapsing-remitting MS, and had a mild degree of disability, as well as an expanded disability status scale (EDSS) (Kurtzke, 1983) score of ≤ 3.5, no relapse or changes in MS modifying medication within the last three months before inclusion, received no pharmaceutical treatment for fatigue, and had no psychiatric diseases, infections, sleep disturbances, drug or alcohol abuse, and no MRI contraindications, including pregnancy. The research project was approved by the local Scientific Ethical Committee of the Capital Region of Denmark [Protocol no.: H-4-2013-182]. Informed written consent was obtained from all participants.

Four patients were excluded from further analyses, because their Beck Depression Inventory II (BDI-II) (Minden et al., 2014) scores indicated severe depression (≥29) (n = 3), or due to missing MRI data (n = 1). Thus, data from 46 patients with relapsing-remitting MS (33 females and 13 males) and 25 healthy volunteers (16 females and 9 males) were used in this study. Demographic and clinical group data of patients and controls are presented in Table 1. The Edinburgh Handedness Inventory was used to evaluate dexterity of participants.

**Table 1:** Clinical characteristics for MS patients and healthy controls.

Subjects	Multiple sclerosis patients n = 46			Healthy controls n = 25				
	Mean	Range	SD	Mean	Range	SD	p – value	
Age	36.1	22–53	8.6	35.8	19–55	10.6	0.872	
Gender** (F:M)	33 : 13	–	–	16 : 9	–	–	–	
EDSS score	1.5	0–3.5	1.3	–	–	–	–	
Disease duration	6.4	0–28	5.2	–	–	–	–	
FSMC total score*	59.9	20–92	21.5	28	19–46	8.2	<0.001	*
FSMC motor score*	29	10–25	10.4	12.9	10–23	3.2	<0.001	*
FSMC cognition score*	30.9	10–48	12.2	15	9–28	5.6	<0.001	*
BDI – II score*	7.7	0–22	6.4	1.5	0–11	2.8	<0.001	*
PSQI score*	5.1	1–18	3.7	3.4	1–5	1.3	0.010	*
ESS score	8.0	2–17	3.9	6.4	0–14	3.9	0.769	
PASAT score	50.3	33–60	7.9	51.1	43–59	5.0	0.089	
SDMT score	54.8	35–89	10.6	56.4	41–70	6.7	0.013	*
EHI score	89.5	85.6–93.4	15.0	94.0	88.6–99.3	9.4	0.186	
9HPT right hand score	15.9	12.8–24.2	2.0	15.7	12.6–19.3	1.8	0.749	
9HPT left hand score	17.6	13.1–26.4	2.2	17.2	14.4–21.5	1.7	0.718	
GM volume unit (mL)	632	512–752	52	657	521–748	70	0.081	
WM volume (mL)	446	333–610	58	466	388–584	55	0.215	
eICV (mL)	1553	1247–1961	147	1595	1330–1850	155	0.357	

Abbreviations:* = p-value < 0.05.

Age = Age in years, BDI-II = Beck Depression Inventory version II, Disease duration = Years since diagnose, EDSS = Expanded Disability Status Scale, EHI = Edinburgh Handedness Inventory, eICV = Estimated Intra Cranial Volume, ESS = Epworth Sleepiness Scale, FSMC total = Fatigue Scale for Motor and Cognitive Functions total score, FSMC motor = FSMC motor score, FSMC cognitive = FSMC cognitive score, FMS = Fatigued MS patients, NFMS = Non-fatigued MS patients, GM = Grey Matter, PASAT = Paced Auditory Serial Addition Test, PSQI = Pittsburgh Sleep Quality Index, SDMT = Symbol Digit Modalities Test, Therapy = In treatment with MS disease modifying drugs or not, 9HPT = Nine Hole Peg Test for right and left hand, WM = White Matter.

**=Ratio.

Using a FSMC_MOTOR_ score of ≥27 as cut-off, 29 MS patients were classified as patients with motor fatigue (FMS patients), and 17 patients were labelled as non-fatigue patients (NFMS patients). The FMS and NFMS groups did not differ regarding the frequency of on-going immunomodulatory therapies. Table 2 summarises the clinical data and scores of relapsing-remitting MS patients with and without motor fatigue.

**Table 2:** Clinical characteristics for motor fatigued MS patients and non motor fatigues MS patients FSMC ≥ 27.

Subjects	Patients with fatigue n = 29			Patients without fatigue n = 17				
	Mean	Range	SD	Mean	Range	SD	p – value	
Age	36.6	25–53	8.1	35.2	22–50	35.2	0.162	
Gender** (F:M)	23 : 6	–	–	10 : 7	–	–	0.143	
EDSS score	2.5	0–3.5	0.7	1.8	0–3.5	1.1	0.014	*
Disease duration	6.0	0–16	4.3	7	1–28	6.3	0.547	
FSMC total score*	72.9	45–92	12.3	37.9	20–67	14.6	<0.001	*
FSMC motor score*	35.9	27–42	5.3	17.4	10–25	5.5	<0.001	*
FSMC cognition score*	37.1	15–48	8.9	20.5	10–42	9.8	<0.001	*
BDI – II score**	10.4	0–22	6.4	3.3	0–10	3.4	<0.001	*
PSQI score	5.7	1–18	4.4	4.2	1–9	1.9	0.197	
ESS score	8.8	3–17	3.6	6.8	2–17	4.3	0.098	
PASAT score	49.0	33–60	8.1	52.8	41–60	4.9	0.083	
SDMT score	54.2	35–71	9.5	56.0	10–89	12.6	0.595	
EHI score	89.8	84.1–95.5	12.7	89.1	81.6–96.5	18.7	0.879	
9HPT right hand score	15.8	12.8–24.2	2.0	16.0	12.9–21.5	2.0	0.851	
9HPT left hand score	17.7	14.3–26.4	2.6	17.1	13.1–19.5	1.4	0.428	
GM volume unit (mL)	623	512–716	51	648	530–752	52	0.420	
WM volume (mL)	439	333–517	51	460	355–610	68	0.559	
eICV (mL)	1521	1247–1689	129	1608	1334–1961	162	0.167	

Abbreviations: * = p-value < 0.05 Age = Age in years, BDI-II = Beck Depression Inventory version II, Disease duration = Years since diagnose, EDSS = Expanded Disability Status Scale, EHI = Edinburgh Handedness Inventory, eICV = Estimated Intra Cranial Volume, ESS = Epworth Sleepiness Scale, FSMC total = Fatigue Scale for Motor and Cognitive Functions total score, FSMC motor = FSMC motor score, FSMC cognitive = FSMC cognitive score, FMS = Fatigued MS patients, NFMS = Non-fatigued MS patients, GM = Grey Matter, PASAT = Paced Auditory Serial Addition Test, PSQI = Pittsburgh Sleep Quality Index, SDMT = Symbol Digit Modalities Test, Therapy = In treatment with MS disease modifying drugs or not, 9HPT = Nine Hole Peg Test for right and left hand , WM = White Matter.

**=Ratio.

### 2.2 Study protocol

All subjects were invited for two separate visits. At day one, participants underwent clinical tests and examinations conducted by a medical doctor. At day two, participants underwent a scan session including functional, structural, dMRI and quantitative MRI scans. Results from the functional MRI are reported in (Svolgaard et al., 2018). All scan sessions were performed in the morning and within the same time frame for all subjects. Here we focus on the dMRI data, while the other data will be reported elsewhere.

### 2.3 Clinical assessment

The participants completed a comprehensive test battery of psychological and neurological examinations. General disability was evaluated by the EDSS. Fatigue was evaluated with the Fatigue Scale for Motor and Cognitive functions (FSMC) (Penner et al., 2009). The FSMC questionnaire was specifically designed to evaluate fatigue in MS. It yields separate scores for motor fatigue and cognitive fatigue, which can be combined into a total fatigue score. Dexterous hand function was evaluated by the Jepsen Taylor Hand Function test (JTHF) and the Nine-Hole Peg Test (9-HPT) (Jebsen et al., 1969, Lamers et al., 2017). Moreover, subjects were screened for depression by Beck Depression Inventory II (BDI-II). Cognitive performance was assessed by the Paced Auditory Serial Addition Test (PASAT) (Bozzali et al., 2013) and Symbol Digit Modality Test (SDMT) (López-Góngora et al., 2015, Possa, 2010). The Pittsburgh Quality of Sleep Index (PSQI) and Epworth Sleepiness Scale (ESS) were applied for sleep quality evaluation and daytime sleepiness respectively (Buysse, 1989, Johns, 1991, Rupprecht et al., 2017).

### 2.4 Magnetic resonance imaging

Whole-brain MRI was performed with a 3.0 Tesla Philips Achieva MRI scanner with a 32-channel head coil (Philips, Best, The Netherlands). The following acquisitions were collected in one session: A structural 3D T1-weighted scan using a magnetization-prepared rapid gradient-echo (MPRAGE) sequence (TR = 6 ms, TE = 2.70 ms, flip-angle = 8°, 0.85 mm isotropic voxels with a field-of-view (FOV) of 245 × 245 × 208 mm), and T2-weighted turbo spin echo sequence (TR = 2500 ms, TE = 270 ms, flip-angle = 90°, 0.85 mm isotropic voxels and a FOV of 245 × 245 × 190 mm). For lesion delineation, a FLAIR image was acquired (TR = 4800 ms, TE = 327 ms, 1 mm isotropic voxels and a FOV of 256 × 256 × 202 mm).

Multi-shell dMRI data were acquired with 2 mm isotropic voxel resolution using a pulsed gradient spin echo (PSGE) sequence with echo planer imaging (EPI) readout. The DWI protocol included three separate shell acquisitions including 6 non-collinear directions with b = 300 s/mm^2^ (TR = 9253 ms, TE = 85 ms, scan time = 1:23 min) and 62 non-collinear directions with b = 1000 s/mm^2^ (TR = 9268 ms, TE = 85 ms, scan time = 10:02 min) and b = 2000 s/mm^2^ (TR = 10861 ms, TE = 98 ms, scan time = 11:41 min). All shells included a single volume with b = 0 s/mm^2^ (b0). Additionally, two datasets each with three b0 images were acquired with both posterior–anterior (PA) and anterior-posterior (AP) phase encodings matching the parameters for both the 1000 s/mm^2^ (scan time = 1:32 min) and the 2000 s/mm^2^ (scan time = 1:48 min) shells, which were used to model and correct for geometrical distortions. The total MRI acquisition time in the study was 1 h and 50 min.

### 2.5 Data analysis of structural MRI

#### 2.5.1 Total lesion number and lesion volume

The processing of the structural MRI was performed in SPM 12 version 6470 (Welcome Department of Imaging Neuroscience, London, UK). The steps included co-registration of the T2-weighted and FLAIR images to the T1-weighted image followed by co-registration to (but not spatial normalized) to standard space using the Montreal Neurological Institute (MNI) atlas. A single observer blinded to all subjects’ clinical characteristics segmented the lesions. The lesions were delineated semi-automatically on FLAIR images using local thresholding technique (Jim 6.0 Xinapse System, Leicester, UK). Corresponding T1- and T2-weighted images were used to verify the lesions. The volume and number of lesions were calculated per subject. To generate a lesion frequency map, we normalized the individual T1-weighted images to MNI space and applied the same non-linear warp to the lesion maps. The normalized lesions maps were then summed across subjects to generate a lesion frequency map.

#### 2.5.2 Brain volumes

Cortical reconstructions and volumetric segmentations measuring GM and WM volumes were conducted using FreeSurfer software (version 5.3.0; http://surfer.nmr.mgh.harvard.edu). The data were processed using a standard pipeline. The technical details of these procedures are previously described elsewhere (Dale et al., 1999, Fischl et al., 1999). In brief, the processing steps include intensity normalization of the T1-weighted images to MNI-space, skull stripping, filtering, segmentation, and surface deformation. Quality assurance of the skull stripping and exactness of the GM and WM outer boundaries were visually inspected by a skilled researcher. Voxels that contained lesions, as defined by the semi-automatic technique as described above, were defined as WM hyper-intensities in the FreeSurfer segmented brain. Using specialized parcellation tools in FreeSurfer, the volumetric data of total intracranial volume (TIV), white matter volume (WMV) and grey matter volume (GMV) were extracted (Desikan et al., 2006).

### 2.6 Diffusion weighted imaging

#### 2.6.1 Pre-processing

Pre-processing of the dMRI data included correction for susceptibility artefacts, head motion, and correction for eddy currents and was performed using the Topup and Eddy tool boxes as implemented in the FSL freeware package (https://fsl.fmrib.ox.ac.uk/fsl/fslwiki/) (Andersson et al., 2003, Smith et al., 2004). The pre-processing pipeline produced a volume-to-volume estimate of root-mean-square movement during the acquisition, which we averaged across all volume-to-volume movements for each participant producing a single estimate of head movement during the acquisition, RMS_MOV._

To account for the different TE and TR parameter settings between shells, each shell was calibrated according to the b0 using the following procedure. Firstly, an overall average b0 image was calculated (averaging the b0 images from the b = 1000 s/mm^2^ and b = 2000 s/mm^2^ acquisitions). Subsequently, a voxel-wise calibration image was calculated by voxel-wise dividing the b0 image of each shell by the overall mean b0 resulting in a shell-specific calibration image. All volumes within each shell were then divided (voxel-wise) by the shell specific calibration image resulting in calibrated dMRI images. The calibrated dMRI dataset was then used for tractography and data analysis.

#### 2.6.2 Anatomical connectivity mapping

The ACM method can be summarized as follows. Initially, the fiber orientation distributions in three-dimensional space was estimated in each voxel. Subsequently, probabilistic tractography was used to sample streamline path based on the voxel wise fibre orientated distribution (FOD) obtained from a local multi-fibre reconstruction model i.e. a spherical deconvolution model. The streamline trajectories were terminated by a given FOD amplitude, to avoid streamlining outside the WM and ensure that the probability maps consisted of voxels with high structural architecture. Thus, the anatomical connectivity of the WM is reflected by restricted diffusion processes in predominant directions, which constitute the strength of connectivity based on the streamline counts.

A critical difference between the present and previous ACM approaches is that only voxels within the same hemisphere were considered, no voxels were “allowed” to travel to the other hemisphere via the corpus callosum or the commissures. Hence, ACM values were derived from unilateral tractography that only considered either the right or the left half of the brain's white matter compartment. This excluded any influence on the lateralization effects coming from the contralateral callosal projecting streamlines.

Tractography was based on a FOD realised using the multi-shell multi-tissue constrained spherical deconvolution (CSD) method as implemented in MRtrix3 (http://www.mrtrix.org/) (Jeurissen et al., 2014). First, using MRtrix3′s 5ttgen, the co-registered structural T1-weighted image was segmented into cortical GM, sub-cortical GM, WM, and cerebrospinal fluid (CSF). CSD response functions were then estimated for GM, WM, and CSF, which was then used to estimate the fibre orientation distribution (FOD) in each voxel allowing for up to 3 distinct fibre orientations (Tournier et al., 2004). The method incorporating multi-shell dMRI with b-value of b1000 and b2000, offers a unique opportunity to model differences in the WM microstructure (Jeurissen et al., 2014).

To generate ACM, we used probabilistic tractography where 300 streamlines were seeded randomly in each WM voxel of the right or left half of the brain. We have previously shown that 300 streamlines are sufficient to yield stable ACM estimates (Lyksborg et al., 2014). Subsequently, the number of streamlines running through each voxel was counted and summarised in a voxel-wise ACM. MRtrix3′s tckgen-function using default parameters were used for tractography. Since we were interested in unilateral anatomical connectivity, the brain was split in the mid-sagittal plane, excluding all streamlines passing the mid-sagittal plane by using an exclusive ROI of the whole midsagittal plan.

#### 2.6.3 Diffusion tensor modelling

Water diffusion within each voxel was also estimated using the diffusion tensor model (Basser, 1995). For each voxel, we calculated fractional anisotropy (FA) and mean diffusivity (MD) for analysing MS lesions and in normal appearing white matter (NAWM) (Bammer et al., 2000, Fink et al., 2010). FA and MD values were calculated from all three shells in subjects’ dMRI space.

#### 2.6.4 Population template

For voxel-wise group analysis of ACM- and DTI-based metrics, we generated a study-specific brain template. This was done using MRtrix3′s population_template function using all FOD images from the study population. Then, to get the generated population template in MNI orientation, we co-registered it to FSL’s MNI FMRIB58_FA_2mm template by rigid body transformation using FSL’s FLIRT. All ACM, FA, and MD maps were then warped to the template using the individual’s transformation matrix obtained from MRtrix3 study population.

For each individual, a NAWM mask was created using the WM segmentation from FreeSurfer, where the manually segmented lesions have been removed. The subject specific NAWM mask was then co-registered to dMRI space. Thereafter, all voxels with FA < 0.3 was removed from the NAWM mask to reduce partial volume effects from transitions between WM, GM and CSF.

We used an atlas-based approach to determine which white-matter voxels belonged to the CST. The extent of CST was defined based on the JHU WM tractography atlas (ICBM-DTI-81 John Hopkins University), which is implemented in the FSL software. This atlas includes a probabilistic atlas of several white matter tracts. All voxels that had a >10% probability of belonging to either left or right CST were included in our CST mask. In our population template space, we made subject-specific masks for bilateral NAWM within the corticospinal tract by intersecting the subject-specific NAWM masks with the corticospinal tract masks from the JHU WM tractography atlas (ICBM-DTI-81 John Hopkins University). We refer to the NAWM within the CST mask as CST-NAWM. In addition, we considered the NAWM outside the corticospinal tract by removing the corticospinal tract from the left and right NAWM masks (referred to as non-CST-NAWM). We then calculated mean ACM, FA, and MD values for left and right CST-NAWM and non-CST-NAWM in each subject. It should be noted that although ACM is mostly tract-specific, anatomical connectivity values of voxels located in the corticospinal tract might be affected by other crossing fibre systems and vice versa but not from streamlines emanating from the other hemisphere, passing through the corpus callosum or anterior or posterior commissures. In addition to the ROI analyses, we also conducted voxel-wise analyses. For the voxel-wise analyses we smoothed the ACM, FA, and MD maps with a 4 mm full-width half-maximum Gaussian kernel to adjust alignment differences between subjects.

To assess lateralized changes in microstructure, we quantified left-right asymmetry calculating a lateralisation index (LI) for mean ACM, FA, MD values and lesion load using the formula LI = 100*(left–right)/(left + right) (Angstmann et al., 2016). Values in the range from −1 to −100 reflect higher values in the right hemisphere, whereas values in the range from 1 to 100 reflect higher values in the left hemisphere.

#### 2.6.5 Statistical analysis

Demographic, clinical and behavioural data were analysed in IBM SPSS (version 24 for Mac, IBM Corp., Armonk, New York, USA). We assessed whether there were any group differences between head movement during the dMRI acquisitions, because they could contribute to any between-group difference in DWI measures. To this end, we compared the average movement estimate, RMS_MOV,_ between HC and MS as well as between FMS and NFMS subgroups using two-samples T-tests in SPSS.

To test our hypotheses, left and right ROI measures were used as dependent variables in repeated measures ANOVAs for between- and within-group analyses. Conditional on significant F-values, pair-wise post-hoc F-tests were performed to elucidate the between-group differences driving the ANOVA results. Further, Pearson's correlations were applied when applicable. We used gender and age as covariates in all models. Analyses of anatomical connectivity accounted for differences in head size by including the total individual ACM count across the whole brain as additional covariate. Finally, voxel-wise analyses of the ACM, FA and MD maps were performed using permutation tests, as implemented in FSL’s Randomise (https://fsl.fmrib.ox.ac.uk/fsl/fslwiki/Randomise). We computed two-sample T-tests comparing healthy controls and patients with MS and contrasting the FMS and NFMS group. Further, EDSS, mean ACM and DTI indices were correlated within the patient group. To account for multiple comparisons, threshold-free cluster enhancement (TFCE) using 5000 permutations was used, which controls for multiple comparisons using family-wise error. P_FWE_ < 0.05 were accepted as significant.

## 3 Results

All participants were right-handers. There were no between-group differences regarding age, gender ratio or handedness (Table 1, Table 2). No differences in head movement during dMRI data acquisition were found between the HC and MS group (T(69) = 0.179, p = 0.674) or between the FMS and NFMS group (T(44) = 0.027, p = 0.870).

### 3.1 Anatomical connectivity of the corticospinal tract

**Fig. 1:**
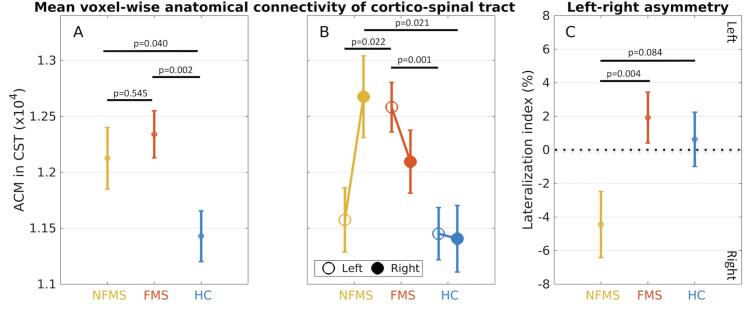
Mean group data of ipsi-hemispheric anatomical connectivity as derived from anatomical connectivity mapping (ACM) of the right and left corticospinal tract (CST). A: The plot shows increased mean ACM values of the corticospinal tract in MS patients without motor fatigue (NFMS group, labelled in yellow) and with motor fatigue (FMS group, labelled in red) compared to healthy controls (HC group, labelled in blue). B: Mean ACM values in left and right CST for each group. The FMS group shows an increase in mean ACM value in the left CST relative to the NFMS and HC groups. The NFMS group shows an increase in mean ACM values in right CST. C: Left-right lateralization of anatomical connectivity in the CST: The NFMS group shows a negative lateralization index, indicating a stronger right-ward asymmetry of ipsi-hemispheric anatomical connectivity in NFMS patients as opposed to FMS patients and healthy controls. (For interpretation of the references to colour in this figure legend, the reader is referred to the web version of this article.)

Both the FMS and NFMS groups had higher mean ACM values in the CST-NAWM compared with healthy controls (Fig. 1A, Table 3). This between-group difference was reflected by a main group effect (F(2,65) = 5.833, p = 0.005; post-hoc FMS > Controls: p = 0.002; post-hoc NFMS > Controls: p = 0.040). We also found lateralized differences in ACM of the CST-NAWM between groups (Side × Group interaction: F(2,65) = 3.983, p = 0.023). Left-right differences in ACM of the CST-NAWM were mainly expressed in MS patients. NFMS patients showed higher mean ACM values in the right relative to the left CST-NAWM, whereas the FMS patients showed higher mean ACM values in the left relative to the right CST-NAWM (Fig. 1B, Table 4). Moreover, mean ACM values in the left CST-NAWM were higher in FMS patients relative to NFMS patients (F(1,41) = 5.682, p = 0.022) and healthy controls (F(1,49) = 12.586, p = 0.001). The NFMS group showed higher mean ACM values in right CST-NAWM relative to controls (F(1,37) = 5.825, p = 0.021), but not relative to FMS patients (F(1,41) = 1.803, p = 0.187).

**Table 3:** MRI diffusion metrics for patients with multiple sclerosis and healthy controls between group comparison.

		Multiple sclerosis patients n = 46			Healthy controls n = 25				
		Mean	SD	95% CI	Mean	SD	95% CI	p – value	
ACM values – Non-CST-NAWM	Left	7200	681	(7026, 7403)	7072	723	(6781, 7300)	0.280	
	Right	7218	668	(7043, 7420)	6975	712	(6695, 7206)	0.083	
	LI(%)	−0.140	2.594	(−1.026, 0.799)	0.679	3.800	(−0.610, 1.868)	0.340	
ACM values – CST-NAWM	Left	12165	1388	(11824, 12564)	11535	1227	(10973, 11954)	0.018	*
	Right	12277	1428	(11873, 12754)	11467	1815	(10801, 11998)	0.017	*
	LI(%)	−0.464	7.006	(−2.946, 2.061)	0.681	10.360	(−2.760, 4.041)	0.611	
FA values – Non-CST-NAWM	Left	0.429	0.014	(0.426, 0.434)	0.437	0.014	(0.432, 0.443)	0.027	*
	Right	0.430	0.013	(0.427, 0.435)	0.438	0.014	(0.433, 0.444)	0.029	*
	LI(%)	−0.098	0.486	(−0.243, 0.043)	−0.067	0.468	(−0.259, 0.129)	0.776	
FA values – CST-NAWM	Left	0.570	0.025	(0.563, 0.577)	0.571	0.021	(0.562, 0.581)	0.851	
	Right	0.570	0.021	(0.564, 0.577)	0.572	0.022	(0.564, 0.581)	0.725	
	LI(%)	−0.054	1.335	(−0.404, 0.311)	−0.078	0.957	(−0.579, 0.391)	0.876	
MD values – Non-CST-NAWM	Left	0.698	0.025	(0.691, 0.705)	0.681	0.017	(0.672, 0.690)	0.004	*
(10^−3^)	Right	0.702	0.024	(0.695, 0.709)	0.684	0.019	(0.674, 0.693)	0.002	*
	LI(%)	−0.274	0.510	(−0.434, −0.116)	−0.193	0.565	(−0.408, 0.023)	0.541	
MD values – CST-NAWM	Left	0.611	0.026	(0.604, 0.618)	0.589	0.025	(0.579, 0.598)	<0.001	*
(10^−3^)	Right	0.627	0.026	(0.621, 0.635)	0.619	0.026	(0.595, 0.614)	<0.001	*
	LI(%)	−1.333	1.170	(−1.652, −1.031)	−1.354	0.776	(−1.761, −0.918)	0.993	

Abbreviations: * = p-value < 0.05, uncorrected CST = Corticospinal tract, eICV = Estimated intracranial volume, LI = Lateralization index, FA = Fractional anisotropy, MD = Mean diffusivity, Non – CST NAWM = Normal appearing white matter hemisphere without CST.

**Table 4:** MRI diffusion metrics for fatigued MS patients and non-fatigued patients between group comparison.

		Fatigued MS patients n = 29			Nonfatigued MS patients n = 17				
		Mean	SD	95% CI	Mean	SD	95% CI	p – value	
ACM values – Non-CST-NAWM	Left	7275	784	(7167, 7590)	7072	449	(6615, 7176)	0.010	*
	Right	7223	775	(7072, 7531)	7209	451	(6772, 7380)	0.252	
	LI(%)	0.339	2.881	(−0.414, 4.481)	−0.959	1.808	(−2.546, −0.036)	0.028	*
ACM values – CST-NAWM	Left	12377	1300	(12039, 13015)	11803	1498	(10900, 12193)	0.022	*
	Right	11942	1212	(11555, 12573)	12848	1618	(11966, 13313)	0.187	
	LI(%)	1.756	6.749	(−0.557, 4.425)	−4.253	5.843	(−7.857, −1.256)	0.004	*
FA values – Non-CST-NAWM	Left	0.431	0.012	(0.426, 0.437)	0.427	0.017	(0.420, 0.435)	0.391	
	Right	0.431	0.011	(0.426, 0.437)	0.428	0.060	(0.422, 0.436)	0.549	
	LI(%)	−0.048	0.471	(−0.231, 0.145)	−0.184	0.513	(−0.441, 0.054)	0.339	
FA values – CST-NAWM	Left	0.569	0.024	(0.560, 0.580)	0.570	0.027	(0.558, 0.584)	0.905	
	Right	0.571	0.016	(0.563, 0.579)	0.568	0.027	(0.559, 0.580)	0.778	
	LI(%)	−0.186	1.476	(−0.657, 0.340)	0.170	1.055	(−0.533, 0.779)	0.499	
MD values – Non-CST-NAWM	Left	0.659	0.025	(0.686, 0.706)	0.702	0.024	(0.688, 0.713)	0.594	
(10^−3^)	Right	0.701	0.026	(0.692, 0.711)	0.703	0.022	(0.689, 0.714)	1.000	
	LI(%)	−0.385	0.566	(−0.583, −0.202)	−0.084	0.335	(−0.324, −0.077)	0.050	*
MD values – CST-NAWM	Left	0.611	0.026	(0.588, 0.620)	0.614	0.091	(0.600, 0.627)	0.658	
(10^−3^)	Right	0.625	0.024	(0.616, 0.644)	0.631	0.029	(0.617, 0.634)	0.591	
	LI(%)	−1.285	1.273	(−1.775,−0.880)	−1.415	1.001	(−1.932,−0.755)	0.964	
Lesion Volume (ml) -NAWM	Left	2.648	2.652	(1.458, 3.837)	3.469	3.931	(1.916, 5.022)	0.402	
	Right	1.303	1.618	(0.617–1.989)	1.901	2.158	(1.005, 2.797)	0.291	
	LI(%)	40.629	14.740	(34.578, 46.680)	34.193	18.400	(26.290, 42.096)	0.199	
Lesion Volume (ml) – CST	Left	0.104	0.179	(0.026, 0.205)	0.105	0.245	(0.004, 0.205)	0.987	
	Right	0.139	0.263	(0.016, 0.263)	0.279	0.419	(0.118, 0.440)	0.172	
	LI(%)	−13.422	70.413	(−39.262, 12.417)	−13.030	66.583	(−46.780, 20.719)	0.985	
Lesion number	Number	54.759	46.900	(38.08, 71.43)	44.529	40.000	(22.74, 66.31)	0.456	

Abbreviations: * = p-value < 0.05, uncorrected CST = Corticospinal tract, eICV = Estimated intracranial volume, LI = Lateralization index, FA = Fractional anisotropy, MD = Mean diffusivity, Non – CST NAWM = Normal appearing white matter hemisphere without CST.

A within-group comparison of mean ACM values between the left and right CST-NAWM only revealed a left–right difference in the NFMS group (p = 0.016), but not in the other two groups (p > 0.148).

Left-right differences in anatomical connectivity were assessed in further detail, comparing the left-right lateralization index of ACM values (ACM-LI) between groups. We found a significant difference in ACM-LI among the three groups (F(2,65) = 3.384, p = 0.040). This between-group difference was driven by a shift towards negative ACM-LI values in the NFMS group, caused by higher mean ACM values in the right relative to the left CST-NAWM (Fig. 1C, Table 4). The NFMS group showed a stronger rightward asymmetry with lower corticospinal ACM-LI values than the FMS group (F(1,41) = 9.564, p = 0.004). A similar trend also emerged when contrasting the ACM-LI data of the NFMS group and healthy controls (F(1,37) = 3.156, p = 0.084).

We conducted a follow-up analysis in which individual EDSS scores were treated as co-variate. Mean ACM value of the NAWM in L-CST still differed between the FMS and NFMS groups, after adjusting for individual differences in EDSS score (F(1,40) = 8.018, p = 0.007). Moreover, the mean ACM values of L-CST NAWM remained increased in the FMS group compared to NFMS group, after controlling for EDSS score (F(1,40) = 4.546, p = 0.039).

We conducted an additional follow-up analysis, which treated the individual BDI scores as co-variate. This analysis only revealed a trend towards CST asymmetry between the FMS and NFMS group (F(1,40) = 3.489, p = 0.069). Further, the difference mean ACM values in L-CST NAWM was no longer significant (F(1,40) = 2.798, p = 0.102).

**Fig. 2:**
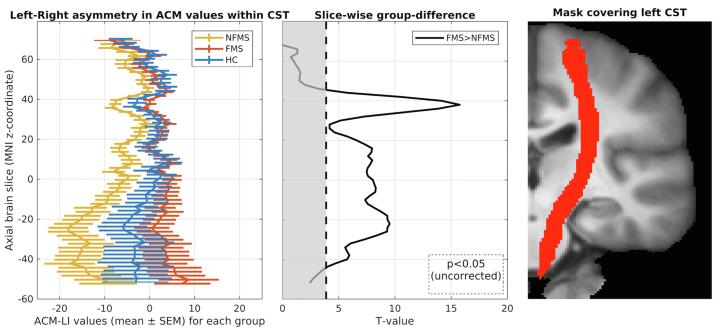
Left-right asymmetry of the ACM values presented for each slice. Left: Slice-wise lateralization index within groups: NFMS group (yellow), FMS group (red) and HC group (blue). The NFMS group differs from the FMS and HC groups, showing a larger right-wards asymmetry caused by increased AC in right hemisphere. The HC group shows no left-right asymmetry, whereas the FMS group shows a trend towards a more left-wards asymmetry. Middle: Slice-wise group difference between FMS and NFMS (white area: p < 0.05). Right: Binary mask of the left CST derived from the probabilistic JHU atlas. ACM: anatomical connectivity mapping. CST: corticospinal tract (CST). LI: laterality index. NFMS: MS patients without motor fatigue. FMS: MS patients with motor fatigue. HC: healthy controls. (For interpretation of the references to colour in this figure legend, the reader is referred to the web version of this article.)

To map where differences in ACM-LI values were expressed along the CST, we performed slice-wise comparisons of ACM-LI values for each axial image slice along the z-axis in stereotactic MNI space. Differences in ACM-LI between the FMS and NFMS groups were found along major parts of the CST (Fig. 2), with slice-wise differences being present in slices covering axial planes form z = -42 to z = 42 (two-sample t-test, uncorrected p < 0.05).

**Fig. 3:**
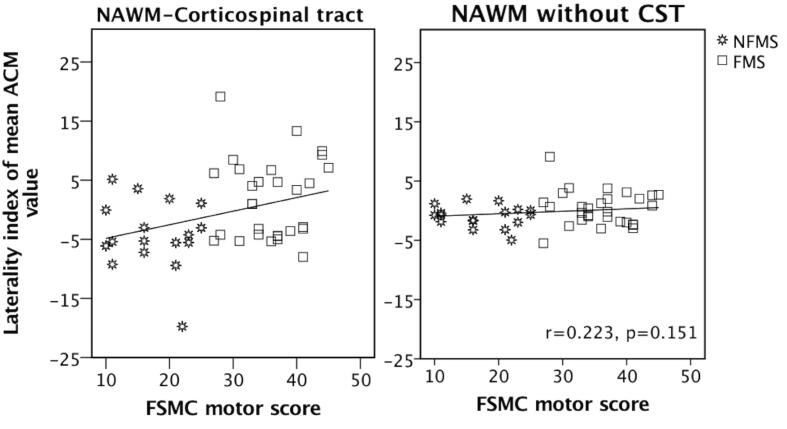
Relationship between the degree of left-right asymmetry of anatomical connectivity in the corticospinal tract (CST) and severity of motor fatigue in patients with relapsing-remitting multiple sclerosis. A: Patients with MS showed a significant positive correlation between the left-right asymmetry of anatomical connectivity in the CST and motor fatigue as reflected by the FSMC_MOTOR_ score. B: Non-significant correlation between non-CST-NAWM LI and FSMC motor score. Both tests were corrected for age, gender and ICV. NAWM-CST: Normal appearing white matter of the corticospinal tract. LI: laterality index. FSMC_MOTOR_: motor subscale of the Fatigue Scale for Motor and Cognitive functions.

The ACM-LI of CST-NAWM scaled positively with the individual FSMC_MOTOR_ scores when all MS patients were considered together (Fig. 3A). Patients showed a positive linear relationship between the ACM-LI values of CST-NAWM and individual FSMC_MOTOR_ scores (r(41) = 0.330, p = 0.031). To investigate the contribution of the left and the right CST-NAWM ACM values to this relationship, we correlated FSMS_MOTOR_ with mean ACM values in the left and right CST-NAWM, separately. There was a trend towards a positive linear relationship with FSMC_MOTOR_ score for mean ACM of the left CST-NAWM (r(41) = 0.259, p = 0.094), while no association was found for mean ACM of the right CST-NAWM (p = 0.336). No associations were found between the ACM-LI of CST-NAWM and the individual FSMC_COGNITIVE_ or FSMC_TOTAL_ scores (p > 0.129), despite of high inter-correlations between the motor and cognitive FSMC score (Penner et al., 2009).

### 3.2 Anatomical connectivity mapping differences in white matter outside the CST

We performed the same set of analyses for the mean ACM of the left NAWM, right NAWM and LI considering all voxels that were located outside the CST (referred to as non-CST-NAWM). No statistically significant main effect or interactions were found (Fig. 3B). However, an exploratory post hoc test revealed an increased left-right asymmetry of ACM in the non-CST-NAWM towards the right hemisphere in the NFMS group compared to the FMS group (F(1,41) = 5.223, p = 0.028) (Table 3, Table 4).

### 3.3 DTI and associations to anatomical connectivity

In general, patients displayed higher MD values than controls. However, no asymmetries were found in the NAWM of CST for mean MD or mean FA (Supplementary Note 1).

Subsequently, correlation analyses were used to explore whether mean ACM values scaled linearly with FA or MD in the combined MS group. In the left hemisphere, mean AC and MD values in CST-NAWM ACM and MD displayed a positive linear relation (r(41) = 0.435, p = 0.004). This was also the case for mean ACM in CST-NAWM and mean MD in non-CST-NAWM (r(41) = 0.342, p = 0.025). In the right hemisphere, a linear relationship was also found between mean ACM and mean MD values in CST-NAWM (r(41) = 0.357, p = 0.019) and a trend towards a significant relationship between mean ACM in CST-NAWM and mean MD in non-CST-NAWM (r(41) = 0.278, p = 0.071). No significant relationships were found between mean ACM and mean FA values in the CST-NAWM (all p > 0.14).

### 3.4 Voxel-wise analysis of dMRI data

Whole-brain voxel-wise analyses showed bilateral decreases in regional FA and widespread increases in regional MD in patients compared to healthy controls. Two clusters in the right frontal lobe showed regional increases in ACM values in MS patients relative to healthy controls (Supplementary Note 2, supplementary Fig. 1).

### 3.5 Lesion load and anatomical connectivity asymmetry

We tested whether the left-right asymmetry of lesion load was associated with the left-right lateralization of mean ACM in MS patients. We found no consistent correlation between ACM-LI of CST-NAWM and the LI for lesion load within the CST (r(46) = 0.151, p = 0.318) and no correlation between ACM-LI of non-CST-NAWM and the LI of lesion load for the non-CST-NAWM (r(41) = 0.216, p = 0.165). However, the ACM-LI of the entire NAWM (including CST) and the LI of global lesion load showed a significant negative relationship (r(41) = -0.345, p = 0.024). Unilateral anatomic connectivity was relatively reduced in the more affected hemisphere expressing a higher lesion load.

### 3.6 Brain volumes and lesion load

We found no differences in white matter volume, grey matter volume or estimated intracranial volume among groups and lesion load between FMS and NFMS patients (Table 1, Table 2, Supplementary Note 3).

## 4 Discussion

### 4.1 Left-right asymmetry of corticospinal connectivity reflects motor fatigue

To the best of our knowledge, this is the first study showing an association between motor fatigue and anatomical connectivity of the corticospinal tracts in patients with a relapsing-remitting type of MS. Using a tractography-derived, voxel-wise metric of mean ipsi-hemispheric anatomical connectivity, patients with motor fatigue showed a relative increase in ipsilateral anatomic connectivity in the left corticospinal tract compared with patients without fatigue and healthy controls. Patients without fatigue only showed an increase of ipsi-hemispheric anatomical connectivity in the right corticospinal tract, while ipsi-hemispheric lateral anatomical connectivity in the left corticospinal tract was unaltered. When considering all patients together, a higher asymmetry of anatomical connectivity of the left relative to the right corticospinal tract scaled positively with the individual severity of motor fatigue.

### 4.2 Regional increases in ipsilateral white-matter connectivity in multiple sclerosis

In addition to the lateralized changes related to motor fatigue, our cohort of mildly disabled patients with relapsing-remitting MS showed an overall increase in ipsi-hemispheric connectivity of the corticospinal tracts compared with healthy controls. The mean ACM of both CSTs did not reflect the magnitude of motor fatigue in the patient group. Voxel-wise comparison of the ACM values at the whole-brain level identified two regions in the right frontal white matter, where patients showed a significant increase in ipsilateral anatomical connectivity relative to controls. No region within the white matter showed a reduction in anatomical connectivity in patients with MS. Intuitively, one might expect that disease-related white matter damage would reduce the anatomical connectivity of white matter voxels in MS and that fatigue may be associated with a reduction in anatomical connectivity.

### 4.3 Biophysical considerations regarding the ACM metrics

For an appropriate interpretation of the ACM metric, it is important to keep in mind that the ACM method does not directly probe the integrity of specific axonal bundle connections. The AC method rather quantifies the probabilistic spread of streamlines within the segmented intra-hemispheric brain network. Hence, MS-induced white-matter damage in the cerebral hemisphere changed the ACM values in the voxels that were assigned to the corticospinal tract via two mechanisms.

One mechanism is driven by local microstructural changes, i.e. lesions and axonal degeneration within CST. Structural damage of the tract of interest, in this study the corticospinal tract, prevents the expansion of streamlines within the tract. This implies that the overall lesion load within one hemisphere should result in reduced ACM values in that hemisphere. In agreement with this assumption, we found a negative relation between the lateralization of ACM in global NAWM and the lateralization of white matter lesion load.

The other mechanism is caused by the fact that crossing fibres are a major cause of reduced anisotropy in the corticospinal tract (Tournier et al., 2012). Disease-related degeneration of corticospinal tract fibres does not only reduce the probability of streamlines, that are seeded in the corticospinal tract, to expand within the corticospinal tract. Neurodegeneration within the CST will also modify streamlines seeded outside the tract. The higher the microstructural damage in the corticospinal tract fibres, the higher is the probability that those streamlines seeded in the crossing fibre tracts (e.g. the longitudinal superior fascicle that cross the corticospinal tract) will expand along the direction of the crossing fibres rather than entering the corticospinal tract. This will lower regional ACM values in the corticospinal tract, because crossing tracts that are relatively spared will attract more streamlines than the damaged corticospinal tract

The impact of crossing fibres on regional ACM in the corticospinal tract also depends on how much the crossing fibre tracts are spared or affected by the disease. Structural damage in those fibre tracts that cross the corticospinal tract may increase ACM of the corticospinal tract, because damage of crossing fibres will increase the probability for streamlines to expand into and along the corticospinal tract rather than staying within the crossing fibre bundles. A relative sparing of crossing fibres may underpin the higher mean ACM values in the corticospinal tract in patients with MS compared to healthy controls. Left-right differences in intra-hemispheric damage of association fibre bundles crossing the corticospinal tract may have mediated the relationship between the asymmetric increase in mean ACM values and the experience of motor fatigue in MS. This aspect is discussed in more detail below.

The DTI-derived microstructural metrics land some support to this notion, but it should be noted that DTI metrics are voxel wise metrics whereas ACM reflect (accumulated) changes align a tract system. Mean ACM in the CST-NAWM correlated positively with mean MD in both, CST-NAWM and non-CST-NAWM. This correlation supports the notion that structural white matter changes within and outside the corticospinal tract may influence ACM values in the corticospinal tract. Increased MD in MS has previously been attributed to pathology indices related to net loss of barriers for molecular motion of water (Cercignani et al., 1999). Hence, widespread MD increases in the ipsilateral white matter may reflect structural white matter alterations that contributed to ACM values in CST-NAWM. Interestingly, a recent DTI study found a correlation between the severity of fatigue and widespread increases in MD in multiple white matter tracts (Batista et al., 2016). We therefore argue that the ACM increase in CST-NAWM does not indicate a real anatomical increase in ipsilateral connectedness between NAWM voxels in the corticospinal tract and all other voxels in the ipsilateral cerebrum. We rather attribute the ACM increase in CST-NAWM to a structural disintegration of fibre tracts crossing the corticospinal tract, causing an increase in streamline counts within the CST.

### 4.4 Comparison with previous ACM studies in multiple sclerosis

While MS patients only showed increases in regional increases in ACM in cerebral white matter, two previous ACM studies demonstrated decreased indices of brain connectedness in MS patients (Bozzali et al., 2013, Lyksborg et al., 2014). In patients with relapsing remitting and secondary progressive MS, we previously reported a widespread reduction in ACM in white matter tracts related to motor control in both MS groups relative to healthy controls (Lyksborg et al., 2014). Patients with secondary progressive MS showed a relative reduction in ACM values in motor-related white matter tracts compared to patients with relapsing-remitting MS (Lyksborg et al., 2014). ACM reduction in these tracts correlated moderately with increased motor disability as reflected by individual EDSS scores (Lyksborg et al., 2014). Another study reported that regional ACM values in corpus callosum, right hippocampus and cerebellum scaled positively with cognitive function as reflected by individual PASAT scores. In both studies, low regional ACM values were associated with dysfunction, presumably because decreases in ACM were most likely caused by a spatial restriction of streamlines by MS-related white matter damage.

Methodological differences may also have contributed to the opposing results between the present and previous ACM studies. One previous study was processed in the Camino toolkit pipeline (www.camino.org.uk), where the estimated fibre directions in each voxel were based on a tensor model, while the present study employed the CSD model. The CSD model deals more efficiently with voxels containing crossing fibres compared to DTI, but also is more prone to elucidate spurious fibre orientation distribution (Tournier et al., 2012). Further, scanner parameter settings during dMRI data acquisition, such as different b-values, may also have an impact in terms of discrepancies between results. In the previous ACM studies, b-values of 1200 s/mm^2^ (Lyksborg et al., 2014) and 1000 s/mm^2^ (Bozzali et al., 2013) were applied. In this study, data were obtained with b-values at both b = 1000 s/mm^2^ and b = 2000 s/mm^2^ to increase angular contrast sensitivity of the fibre response functions (Jeurissen et al., 2014). It is possible that these differences in the acquisition and modelling of the dMRI data resulted in different sensitivity across studies to the impact of crossing fibres on the resulting regional ACM values.

Taken together, the previous ACM studies and our present ACM study in patients with MS show that disease-related changes in regional ACM values are determined by two mechanisms which can be expected to have opposing effects on the direction of regional ACM changes. One mechanism is related to changes in fibre microstructure in the tract of interest and the other mechanism is related to microstructural changes in tracts that path through the tract of interest (i.e., crossing fibres). Both mechanisms may be expressed simultaneously, yet to a variable degree across patients due to inter-individual variations in the regional expression of tract-specific pathology. Any of the two mechanisms may prevail at different stages or in different phenotypes of the disease and may play a more prominent role depending on methodological aspects related to dMRI data acquisition and modelling. It is also possible that both mechanisms are present and may neutralize each other. This may explain that regional abnormalities in MD and to a lesser degree in FA were far more widespread in cerebral white matter in the present study than regional changes in ACM values.

### 4.5 What is the link between altered ACM in corticospinal tract and motor fatigue?

In the previous sections, we attributed the increase in anatomical connectivity in the corticospinal tract in MS patients to a disintegration of fibre tracts that cross the corticospinal tract. A disintegration of fibre tracts that cross the corticospinal tract is also a plausible mechanistic explanation why an increase in mean anatomical connectivity in the CST-NAWM was associated with the patients’ experience of motor fatigue. It is worth to bear in mind that a lateralized rather than a general increase in anatomical connectivity scaled with motor fatigue. Patients suffering from motor fatigue showed abnormal high anatomic connectivity values in the left dominant corticospinal tract. Patients who had no motor fatigue only showed increased connectivity values in the right non-dominant corticospinal tract. In other words, the asymmetry of left and right corticospinal connectivity differed significantly between MS groups, with the NFMS group displaying less structural alteration in the left dominant relative to the right non-dominant corticospinal tract relative to the FMS group.

Asymmetrical involvement of the corticospinal tract has previously been explored in patients with MS. Increased asymmetry as measured by radial diffusivity (DTI) and magnetization transfer ratio (MTR) was reported in patients with MS, while no asymmetry was found for other DTI-based metrics such as FA or MD (Reich et al., 2007). Accordingly, we found no left-right asymmetry for FA or MD in CST-NAWM. Our ACM study significantly extends previous work, showing a more symmetrical alteration of anatomical connectivity in MS patients experiencing motor fatigue than in those without fatigue. Our results further suggest that an abnormal increase of anatomical connectivity in the left CST-NAWM, but not in the right CST-NAWM may be related to the experience of fatigue. This hypothesis is further substantiated by the observation that a lateralization of anatomical connectivity increases in the right and left CST-NAWM scaled positively with the severity of motor fatigue. The higher the ACM values in left CST-NAWM relative to right CST-NAWM, the more patients suffered from motor fatigue. Inspection of the axial brain slices that covered the cerebral hemispheres showed that healthy controls expressed a lateralization of ACM values in the CST-NAWM that fell in between the two MS groups. Relative to healthy controls, MS patients with motor fatigue showed more lateralization of corticospinal ACM values towards the left hemisphere and MS patients without fatigue showed more lateralization of corticospinal ACM values towards the right hemisphere (Fig. 2). We hypothesize that MS patients with motor fatigue show a more left-lateralized disintegration of white matter tracts crossing the corticospinal tract, whereas MS patients without motor fatigue show the opposite pattern with a more right-lateralized disintegration.

### 4.6 Other methodological considerations

Since our analyses accounted for age and there was no difference in head motion parameters between groups, we are confident that the present results were not confounded by age related effects or between-group differences in head motion. Since patients with fatigue are frequently suffering from major depression, we excluded four patients who either had BDI scores indicating severe depression or in whom the BDI score was missing. We also performed a follow-up analysis, which treated the individual BDI scores as co-variate of no interest, the differences between the FMS and NFMS groups were no longer significant. But this does not indicate that depression was the factor driving the ACM differences in the NAWM of the CST between the FMS and NFMS groups. At the symptom level, it is impossible to disentangle minor or moderate symptoms of depression from fatigue, because clinical depression scales contain items that correspond to the core symptoms of fatigue. This implies that MS patients suffering from fatigue will always have higher depression scores than MS patients without fatigue. It is thus not surprising that the inclusion of the BDI score weakened the magnitude of ACM differences between FMS and NFMS patients. This clinical overlap between depression and fatigue renders it inherently difficult to dissociate brain correlates of fatigue from those of depression.

### 4.7 Conclusion and future avenues

Whatever the underlying mechanism may be, our results suggest that abnormal lateralization of connectivity-based properties of the corticospinal tract may constitute a promising MRI-based marker for motor fatigue in MS. The cross-sectional study design did not allow us to assess how the lateralization of corticospinal connectivity changes over time and impacts on the experience of fatigue. Further, we only focused on microstructural measures. Additional measurements of functional connectivity within the corticospinal tracts would have facilitated the interpretation of the present findings. A recent study assessed left-right asymmetry of brain excitability with transcranial magnetic stimulation of the motor cortex and reported that asymmetric corticospinal excitability scaled with disease related symptoms in patients with multiple sclerosis (Chaves et al., 2019). Finally, brain pathology can influence the ACM metric in a complex way. The regional ACM values in a tract of interest are not only altered by an accumulated structural damage along the tract itself but also of tracts crossing through the tract of interest. Therefore, dMRI methodologies that can disentangle these mechanisms would greatly facilitate the interpretation of future ACM studies. Recent progress in dMRI can capture physiological dispersion in fibre orientation already at the data-acquisition level (Andersen et al., 2020) and may allow to objectively estimate the impact of crossing fibre tracts on regional ACM values.

Future studies, featuring a longitudinal study design, more advanced dMRI acquisition protocols that resolve crossing fibres, and additional measurements of functional corticospinal connectivity, are warranted to clarify how lateralized changes of connectivity-based microstructural properties of the corticospinal tract contribute to motor fatigue in MS.

## Declaration of Competing Interest

The authors declare that they have no known competing financial interests or personal relationships that could have appeared to influence the work reported in this paper.
