# Supplementary material for: Motor fatigue is associated with asymmetric connectivity properties of the corticospinal tract in multiple sclerosis
Source: Neuroimage Clin. 2020 Aug 25;28:102393. doi: 10.1016/j.nicl.2020.102393 (PMC7490847; doi:10.1016/j.nicl.2020.102393)
Supplement: Supplementary data 1 [file mmc1.docx]

**Supplementary results**

**Motor fatigue is associated with lateralized changes in connectivity properties of the corticospinal tracts in multiple sclerosis**

Christian Bauer, Tim B. Dyrby, Finn Sellebjerg, Kathrine Skak Madsen, Olivia Svolgaard, Morten Blinkenberg, Hartwig Roman Siebner^*^, Kasper Winther Andersen^*^

* These authors contributed equally as senior authors.

**Corresponding authors**

Hartwig Roman Siebner, MD, DMSci

Danish Research Centre for Magnetic Resonance (DRCMR), Centre for Functional and Diagnostic Imaging and Research, Copenhagen University Hospital Hvidovre, Kettegaard Allé 30, 2650 Hvidovre, Denmark.

E-mail: [h.siebner@drcmr.dk](mailto:h.siebner@drcmr.dk)

**Note 1. DTI of the CST and non-CST**

We tested for between-group differences in mean FA and MD within the left and right CST-NAWM as well as differences in left-right asymmetry for FA and MD in the CST-NAWM as reflected by the LI (i.e. FA-LI and MD-LI). In contrast to the differences revealed by the AC mapping, mean FA and MD did not express between-group differences in left-right asymmetry. There were no statistical between-group differences in unilateral FA values and FA-LI (ps>0.3). Mean MD was significantly different among groups within the CST-NAWM caused by higher mean MD in patients than in healthy controls. Repeated measures ANOVA revealed main-effect of group (F(1,65)=8.137, p=0.001) and a trend towards a main effect of hemisphere (F(1,65)=3.385, p=0.070), but no group-by-hemisphere interaction (p>0.935) for CST-NAWM MD. Post hoc tests revealed increased MD in the FMS and NFMS groups compared to HC in both the left and right CST-NAWM (p<0.001), but no differences between the FMS and NFMS groups (p>0.591).

Left-right asymmetry in mean MD and FA did not differ between groups in non-CST NAWM. The respective ANOVAs showed no main effect of group for FA-LI and MD-LI in the CST-NAWM (p>0.142). Both the FMS and NFMS patients displayed higher mean MD values in right and left non-CST NAWM than healthy controls (main effect of group (F(2,65)=4.807, p=0.011; all post hoc tests: p<0.025). There were no significant main effect of hemisphere (F(1,65)=0.572, p=0.452) or group-by-hemisphere interaction (F(2,65)=2.070, p=0.134). For mean FA, a trend towards main effect of group (F(2,65)=2.985, p=0.057) was found due to higher mean FA values in non-CST NAWM in HC relative to patients (Table 3). There were no main effect of hemisphere and no group-by-hemisphere interaction for mean FA (p>0.096).

**Note 2. Voxel-wise analysis of diffusion MRI data**

The results from voxel-wise analyses of AC, FA, MD and lesions are summarized in Supplementary Fig. 1. The topography of significant WM increases in MD corresponded to the spatial distribution of MS lesions as evidenced by a voxel-wise frequency map of MS lesions. In contrast to the more widespread between-group differences in MD, voxel-wise differences in FA and in particular in AC values were more concise. Two clusters in the right frontal lobe showed increased AC values in patients relative to controls. The first cluster peaked at MNI coordinates x=18, y=36, z=40 (T=4.83, p=0.007, 519 voxels) and the second cluster at x=18, y=4, z=58 (T=4.39, p=0.015, 139 voxels). These clusters covered parts of the anterior thalamic radiation (ATR), inferior fronto-occipital fasciculus, corticospinal tract, superior longitudinal fasciculus, forceps major and forceps minor based on the JHU tractography atlas. No clusters showed significantly lower AC values in the patient group relative to healthy controls. Four clusters in the cerebellum showed a positive linear relationship between regional AC values and individual EDSS score with peaks at MNI coordinates, x=-32, y=-48, z=-40 (T=3.87,p=0.040, 16 voxels), x=-12, y=-46, z=-32 (T=3.56 p=0.039, 20 voxels), x=-10, y=-56, z=-24 (T=3.23, p=0.047, 4 voxels), x=-10, y=-30, z=-26 (T=3.26, p=0.049, 2 voxels).

**Note 3. Brain volumes and lesion load**

Total intracranial volume (eICV), GMV and WMV were calculated for all subjects (Tables 1 and 2). Using age and sex as covariates, we found no between-group differences in eICV (p=0.26), WMV (p=0.17), or GMV (p=0.09) between patients and healthy controls. There was a trend towards a higher eICV in the NFMS group relative to the FMS group (p=0.052), but no difference in WMV (p=0.23) or GMV (p=0.11).

In the entire patient group, mean total number of WM lesions was 51 (range: 9 - 246, median 33), and mean total lesion volume was 5.8 ml (range: 0.3ml - 30.7ml, median 2.8 ml). Lesion volume and number of lesions in the entire WM and the segmented CST are given in Table 4, considering the right and left WM separately. No differences in total lesion load were found in the left and right total WM or corticospinal tract between FMS and NFMS patients (p>0.170). Likewise, global lesion number did not differ between groups. We also tested for a left-right asymmetry in lesion load. To this end, we calculated the LI of mean lesion volume within the CST and the remaining WM (non-CST), respectively. No differences in lesion load lateralization were found between the FMS and NFMS groups in CST (F(1,44)=0.000, p=0.985) and in non-CST (F(1,44)=0.169, p=0.199) (Table 4).


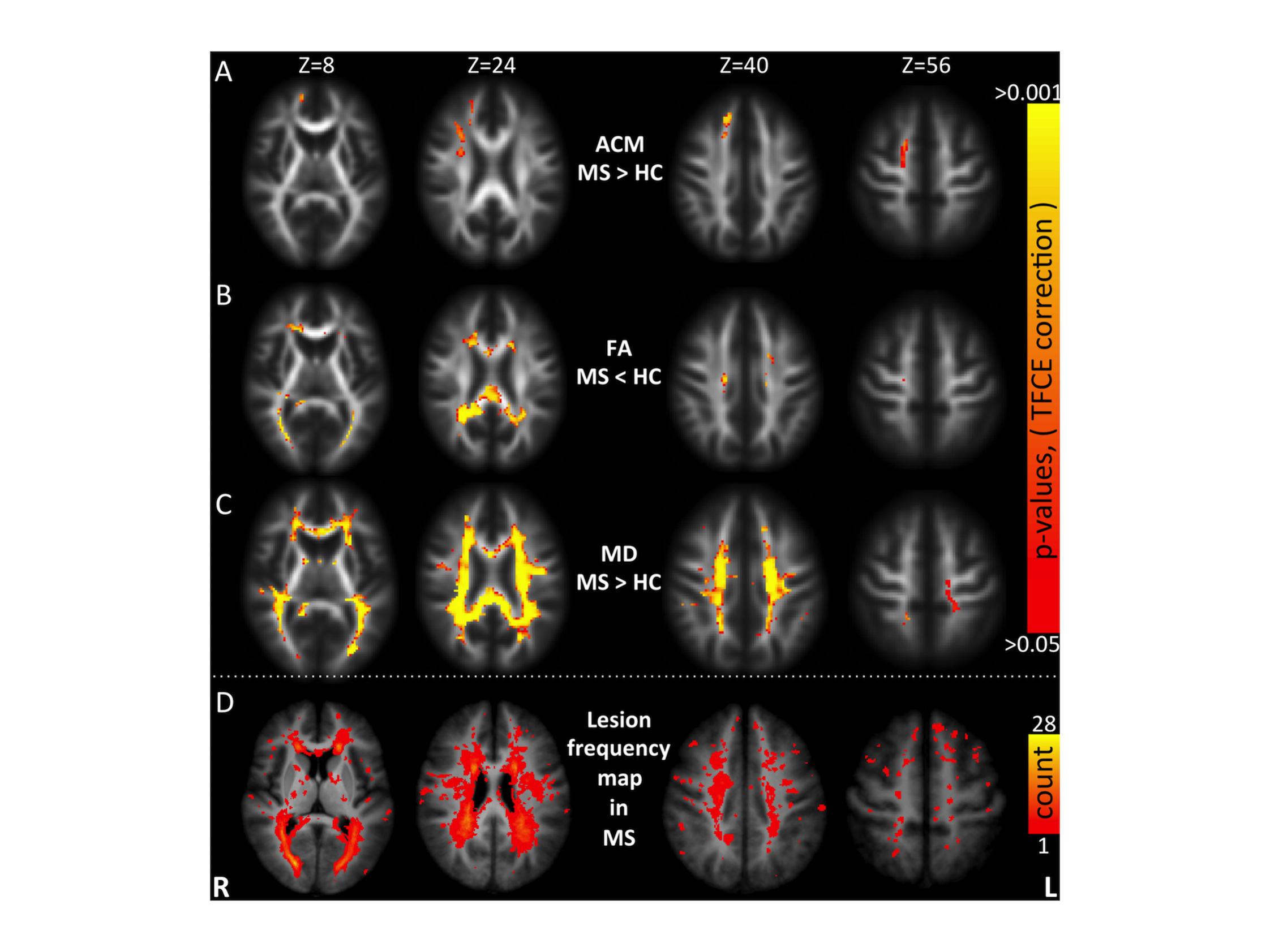
Figure 1.

Figure 1: Statistical parametric maps (SPMs) showing voxel-wise differences in regional ACM, FA, and MD (A-C) and a voxel-specific lesion frequency map. SPMs were thresholded at p<0.05 using the TFCE method to correct for multiple comparisons. (A) Increased ACM among MS compared to HC in right frontal lobe. (B) Increased bilateral FA values among HC compared to MS. (C) Widespread MD increases in MS compared to HC. (D) Lesion frequency map, indicating where lesions are commonly located in the combined MS group. (A-C) is overlaid on the group average FA map, while (D) is overlaid on a group average T1 map. TFCE =Threshold-free cluster enhancement
